# Supported Planar Bilayers for the Formation of Study of Immunological Synapses and Kinapse

**DOI:** 10.3791/947

**Published:** 2008-09-15

**Authors:** Santosha Vardhana, Michael Dustin

**Affiliations:** Helen and Martin S. Kimmel Center for Biology and Medicine at the Skirball Institute of Biomolecular, New York University - NYU

## Abstract

Supported planar bilayers are powerful tools that can be used to model the molecular interactions in an immunological synapse.   To mimic the interactions between lymphocytes and antigen presenting cells, we use Ni2+-chelating lipids to anchor recombinant cell adhesion and MHC proteins to the upper leaflet of a bilayer with poly-histidine tags. Planar bilayers are generated by preparing lipid, treating the glass surfaces where the bilayer will form, and then forming the bilayer in a specialized chamber containing a flow-cell where the lymphocytes will be added.  Then, bilayers are charged with Ni and his-tagged recombinant proteins are added.  Finally,  lymphocytes are injected into the flow cell and TIRF microscopy can be used to image synapse formation and the mechanisms that control T cell locomotion, sites of receptor sorting,  and sites of receptor degradation.

**Figure:**
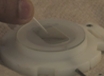


## Protocol

### 1. Preparing the lipids

In order to link proteins to the upper leaflet of a bilayer, we use Ni^2+ ^chelating lipids, which anchor recombinant proteins with poly-histidine tags. Certain cell types may adhere non-specifically to Ni^2+^-coated bilayers, but T-cells generally do not, making this method optimal for studying T cell activation.
The lipid bilayer solution is made by preparing a solution of 20% Ni^2+ ^chelating lipid and 80% phosphatidylcholine in the appropriate amounts in a glass tube with 1-2 ml of chloroform-methanol.
  Evaporate the solvent under a stream of nitrogen gas in a warm (30-37°C) water bath. This usually takes around 15 minutes.
  Remove any remaining solvent under high vacuum, using a lyophilizer for 90 minutes.
Dissolve the lipids in 2% N-octyl-glucoside detergent in Tris-saline buffer, which creates a solution of mixed detergent/phospholipid micelles. The final phospholipid concentration should be 0.4 mM. To form liposomes, dialyze this solution against 3 changes of Tris-saline to remove detergent and form liposomes. We usually work with volumes on the order of 1 ml with 6 mm diameter Spectra/por #2 tubing with a cut off around 10 kDa. We are careful to exclude any air bubbles when clamping the tubing. We typically sterile-filter this solution and do the dialysis under clean conditions, handling it using 70% ethanol sterilized gloves.
This solution should be crystal clear. It is stored under argon gas to prevent oxidation. If the solution is turbid, then multi-walled vesicles have been generated, and you will need to start again.

### 2. Determining how much ICAM-1 and MHC are deposited on the planar bilayer

In order to set up the experiment, it is first necessary to determine how much ICAM-1 and MHC are deposited on a given surface area of Ni^2+^ chelating bilayer at a given concentration of soluble protein. To do this, deposit bilayers on 5 micrometer diameter glass beads, incubate the glass beads with protein solutions under conditions that approximate those in the flow cells used for imaging, and read out the density of bound protein by a flow immunofluorometric assay.
FITC-labeled antibodies with known numbers of fluorescein per molecule are used to detect the surface-bound proteins. Fluorescein standard beads are used to calibrate the flow microfluorimeter.
We will establish conditions that approximate those on antigen-presenting cells with 200 molecules/μm2 of ICAM-1 and 0.2-20 molecules/μm2 of I-Ek-MCC91-103 complex.

### 3. Cleaning the glass cover slips for forming planar bilayers

Planar bilayers form spontaneously when the liposomes fuse to glass, but only if the glass is properly cleaned.
When working with Piranha, we wear full protective clothing, including an acid apron and heavy, acid resistant gauntlets. Carefully segregate the waste from organic wastes and dispose of properly.
To prepare the acid Piranha solution, add 75 ml of concentrated sulfuric acid to a dry beaker, and carefully add 25 ml of 30% hydrogen peroxide. The solution rapidly heats up to greater than 100°C.
Immerse the dry coverslips in the solution for 15 minutes. Remove from the solution and rinse in purified water for a minute on each side. Dry the coverslips using a vacuum source to drain off the purified water. Allow the Piranha solution to cool, and add to the Piranha waste container, which is left with the cap loose, well marked in the fume hood. When all of the liposomes, coverslips, and proteins are prepared, the immunological synapse is ready to be formed.

### 4. Forming the bilayers

To form the bilayers, our lab uses Bioptechs FCSII chambers, which have the advantages of integrated heating. The FCSII system is on a stainless steel base that clamps together a microfluidic manifold with a 0.5 mm top gasket, a microaqueduct slide coated with indium tin oxide for heating on one surface, with fluidic groove on the other side, a dust-free 0.25 mm gasket that defines the height of the flow chamber and the Piranha cleaned 40 mm round coverslip on which the bilayer is formed.
While the cover slip, upon which the bilayer is formed, needs to be highly hydrophilic and clean, the microacqueduct slide actually works better if it's more hydrophobic. Making the microaqueduct more hydrophobic is acheived by treating it with 1% HSA for 60 minutes at room temperature, then washing with pure water and drying completely after this conditioning process, as well as between uses. The coating of denatured proteins left by this procedure allows 1 μl of liposome suspension to form a round, hemispherical drop that is >0.25 mm high.
To assemble the flow cell and form planar bilayers, place the manifold, with the tubes directed upwards, upon a flat surface. Then, place the 0. 5 mm gasket with holes positioned over the fluidic tubes, making sure that it is seated flat. Gently place the microaqueduct side onto the gasket with the fluidic channels facing up, and make sure it seats flat without applying any downward pressure, until verifying that the holes in the slide align with the microfluidic tubes. Lay the dust-free 0.25 mm gasket with a rectangular cut out on top of the microaqueduct slide so that the channel is lined up with the fluidic channels and there are no major air spaces.
Place up to 5 x 1 μl drops of liposome suspension on the surface of the microaqueduct slide in a pattern such that the center-to-center distance between each drop is 2.5 mm. The 5 liposome drops may have different compositions, but in this case we will only form two bilayers, one with no Ni^2+^+ chelating lipids and one with 10% Ni^2+^+ chelating lipids.
Once these drops are in place, carefully place the cover slip down on the surface in one motion. Clamp onto the base as quickly as possible. The liposome drops should make contact with the cover slip. This contact should not be broken, since the bilayers have probably already formed; any disruptions may result in defective bilayers.
It is important to remember to mark the positions of the bilayers with a few small ink spots on the microacqueduct slide to aid positioning of the coverslip on the microscope. Wait 20 minutes to make sure the system has equilibrated. Transfer all the solutions using a 20 ml syringe connected to about 20 cm of flexible tubing and a 3-way stopcock. Connect another port of the stopcock to the flow cell by a short length of tubing to minimize the dead volume between the stopcock and the flow cell. Fill the syringe with HEPES-buffered saline containing 2 mM MgCl_2_, 1 mM CaCl_2_, 5 mM glucose and 1% human serum albumin.
Prime the tubing and stopcock so there are no air bubbles. Connect this to the flow cells and push through 5 ml in one motion while watching to make sure that no air bubbles pass over the bilayer. Now, you will have your bilayers.

This video article supports "Hunter to Gatherer and Back: Immunological Synapses and Kinapses as Variations on the Theme of Amoeboid Locomotion" by Michael L. Dustin, Annual Review of Cell & Developed Biology, Volume 24, 2008.

